# Real-Time Physical-to-Virtual Scene Reconstruction for Dynamic Manufacturing Systems via Semantic—Spatial Mapping

**DOI:** 10.3390/s26206399

**Published:** 2026-10-10

**Authors:** Songling Tian, Jianwen Gu, Lei Pan, Xiaoqian Qi, Peng Wang, Junjun Han

**Affiliations:** 1 School of Control and Mechanical Engineering, Tianjin Chengjian University, Tianjin 300384, China; tiansl201911@tcu.edu.cn (S.T.); gjw201411@163.com (J.G.); panlei4089@163.com (L.P.); qixiaoqian@tju.edu.cn (X.Q.); 2 International Institute for Innovative Design and Intelligent Manufacturing, Tianjin University in Zhejiang, Shaoxing 312000, China; 3 Ningbo Hilectro Precision Machinery Manufacturing Co., Ltd., Ningbo 315801, China; hjj@mail.haitian.com

**Keywords:** manufacturing digital twins, point-cloud processing, semantic–spatial representation, CAD model retrieval, lightweight neural network

## Abstract

Real-time reconstruction of virtual scenes in response to changing physical conditions remains a challenge in manufacturing digital-twin systems. During manufacturing operations, changes in object positions, orientations, and spatial arrangements require the virtual scene to be updated to reflect the evolving physical environment. Here, we present a physical-scene-driven semantic–spatial mapping framework to support real-time scene reconstruction in dynamic manufacturing systems. The framework integrates visual object detection, point-cloud-based spatial estimation, digital asset retrieval, and virtual scene updating. A lightweight detector identifies physical objects and provides semantic information for spatial processing, while object-level point clouds are used to estimate their positions and orientations. The resulting semantic and spatial information is associated with predefined computer-aided design (CAD) models and used to update corresponding virtual objects in a Unity environment. To improve the efficiency of semantic perception, we develop SF-Lite-YOLOv8n by integrating C2f_Star, Spectral-SPPF, and Detect_Efficient. On the MVTec ITODD dataset, the detector achieves an mAP@0.5 of 0.940 with 1.98 million parameters and 6.2 GFLOPs, at a measured inference speed of 83 frames per second. In experiments involving 20 self-collected manufacturing scenes, the integrated perception and mapping pipeline achieves mean position and orientation errors of 2.7 mm and 1.9°, respectively, with a processing latency of 146 ms, excluding Unity rendering. These results demonstrate the potential of combining lightweight semantic perception and point-cloud-based spatial estimation to support the reconstruction and updating of virtual manufacturing scenes.

## 1. Introduction

The rapid development of intelligent manufacturing has accelerated the transition from isolated automation systems towards interconnected cyber–physical production environments. Among emerging technologies, digital twins have attracted considerable attention because they provide a mechanism for representing, monitoring and interacting with physical manufacturing systems through their virtual counterparts [1]. By maintaining a dynamic relationship between physical entities and digital representations, digital twins enable applications including production optimization, process monitoring and intelligent decision-making [2].

Despite significant progress, the practical deployment of manufacturing digital twins remains challenging. A fundamental requirement of a digital twin is the establishment of a reliable correspondence between physical objects and their virtual representations [3]. Although modern manufacturing processes generate accurate CAD models through computer-aided design and engineering systems, transferring these predefined digital resources into a virtual environment that reflects the current physical scene still requires substantial manual intervention [4]. This limitation becomes increasingly significant in flexible manufacturing environments, where production layouts, component arrangements and operational conditions may change frequently [5]. Under such circumstances, a virtual scene configured in advance may gradually become inconsistent with the evolving physical environment. Therefore, enabling efficient physical–virtual correspondence and updating virtual scenes in response to changing manufacturing conditions remain important challenges for digital-twin systems.

Current digital-twin construction approaches generally follow a virtual-model-centered paradigm. In such workflows, digital assets are regarded as predefined representations, while human operators are responsible for selecting appropriate models, defining spatial relationships, and configuring virtual scenes according to observations of the physical environment. Although effective in structured applications, this paradigm relies heavily on manual intervention to establish and maintain the relationship between physical and virtual spaces. Such dependence can limit the efficiency and scalability of digital-twin deployment, particularly in manufacturing environments containing diverse components and frequently changing configurations.

A fundamental question therefore arises: can physical objects themselves become active participants in digital-twin scene reconstruction rather than being manually represented in the virtual environment? Addressing this question requires a transition from virtual-first modeling towards physical-scene-driven interaction. Unlike unknown objects in open environments, manufacturing components often have corresponding digital models stored in engineering databases or digital asset libraries [6,7]. Therefore, the central challenge is not necessarily to reconstruct the complete geometry of every object from the physical world, but to establish an efficient mechanism through which physical objects can be identified and associated with existing digital representations, while their spatial states are estimated from current observations.

Inspired by this observation, we introduce a physical-scene-driven interaction paradigm for manufacturing digital twins. The central idea is to regard physical objects as active information sources that connect real-world manufacturing scenes with virtual environments. In this paradigm, object identity and spatial configuration constitute two complementary dimensions of scene understanding. Semantic perception determines which digital asset corresponds to a physical object, while spatial perception estimates the position and orientation required to align the digital representation within the virtual environment [8,9]. Their integration allows observations of the physical manufacturing scene to provide direct input for virtual scene reconstruction and updating. When the physical configuration changes, newly acquired semantic and spatial information can be used to revise the corresponding virtual object states, reducing the need for repeated manual scene configuration.

To support this paradigm, an efficient semantic perception module is required. Unlike conventional industrial inspection tasks that primarily focus on recognition accuracy, digital-twin scene reconstruction also requires computationally efficient perception to support frequent scene observation and timely virtual updates [10]. Excessive model complexity may introduce additional processing latency and affect the responsiveness of the physical–virtual interaction process. Therefore, this work develops an interaction-oriented lightweight perception approach based on YOLOv8n to provide an efficient semantic interface between physical objects and digital assets [11]. The detector is integrated with point-cloud-based spatial estimation, enabling object identity and geometric state to be used together for physical-to-virtual mapping.

In this study, we propose a physical-scene-driven semantic–spatial mapping framework to support virtual scene reconstruction in dynamic manufacturing environments. By integrating object recognition with three-dimensional spatial estimation, the framework establishes correspondence between physical objects and their virtual representations, enabling virtual scenes to reflect observed manufacturing configurations and accommodate changes in physical object states.

The main contributions of this work are summarized as follows:

(1) We introduce a physical-scene-driven interaction paradigm for manufacturing digital twins, in which physical objects serve as sources of semantic and spatial information for establishing physical–virtual correspondence and supporting scene reconstruction.
(2) We develop SF-Lite-YOLOv8n, an interaction-oriented lightweight object detection model, and integrate it with point-cloud-based spatial analysis to provide efficient semantic and geometric information for object-level mapping.
(3) We implement a physical-to-virtual scene reconstruction framework in Unity and evaluate its detection performance, spatial mapping accuracy, and processing latency using a public benchmark dataset and self-collected manufacturing scenes.

The remainder of this paper is organized as follows: Section 2 introduces the proposed physical-scene-driven interaction framework and describes its overall concept and implementation strategy. Section 3 presents the interaction-oriented semantic–spatial perception approach, including the optimized semantic perception module and spatial-state estimation method. Section 4 describes the manufacturing digital-twin implementation and system integration, followed by experimental validation of semantic perception and physical–virtual mapping. Section 5 discusses the experimental findings, the role of semantic–spatial correspondence in scene reconstruction, and the limitations of the current framework. Finally, Section 6 concludes the study.

## 2. Physical-Scene-Driven Semantic–Spatial Mapping Framework

### 2.1. Concept of Physical-Scene-Driven Interaction

Manufacturing environments are subject to changes in component arrangements, material flow, and operating conditions. To maintain consistency between physical manufacturing systems and their virtual representations, a digital twin should be capable of reflecting the observed physical configuration and accommodating changes in object states. However, conventional digital-twin construction often relies on manually selecting digital assets and configuring their positions and orientations in the virtual environment. This process can become time-consuming when manufacturing scenes contain multiple components or undergo frequent changes.

**Figure 1:**
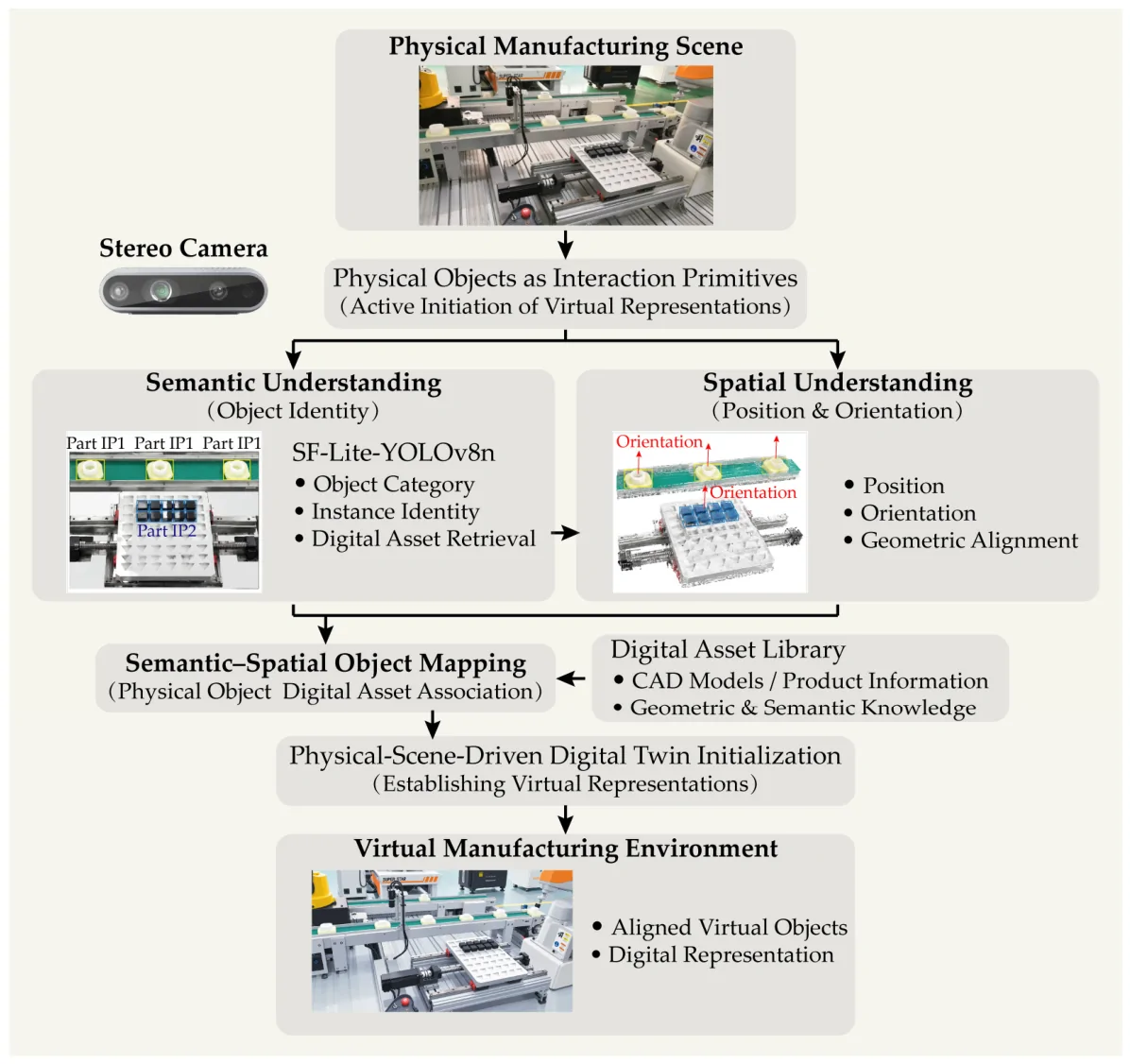
Conceptual framework of the proposed physical-scene-driven semantic–spatial mapping approach for manufacturing digital twins.

To address this limitation, this study introduces a physical-scene-driven semantic–spatial mapping framework for manufacturing digital twins, as illustrated in Figure 1. Rather than treating physical objects as passive entities that require manual representation, the proposed framework uses observations of physical objects to establish their correspondence with virtual assets. The framework focuses on identifying physical objects, estimating their spatial states, and associating them with predefined digital models to support virtual scene reconstruction.

The proposed framework is based on the availability of established computer-aided design (CAD) models for many standardized manufacturing components. Accordingly, the objective is not to reconstruct the complete geometry of each object from sensor data, but to identify the corresponding digital asset and determine its spatial relationship with the physical object. This allows existing geometric resources to be reused while reducing the manual effort required for virtual scene construction.

The framework establishes physical–virtual correspondence through two complementary information dimensions: semantic identity and spatial state. Semantic information identifies the category of a physical object and provides an index for retrieving its corresponding CAD model from a predefined digital asset library. Spatial information describes the object’s three-dimensional position and orientation, providing the geometric constraints required to align the retrieved model in the virtual environment. By integrating these two information dimensions, physical observations can drive the reconstruction of virtual scenes that represent the observed manufacturing configuration.

When changes in the physical arrangement or spatial states of components are observed, the semantic–spatial mapping process provides a basis for updating the corresponding virtual representations. The framework therefore extends beyond one-time manual scene configuration by establishing a perception-driven pathway from physical observations to virtual scene reconstruction and potential subsequent updating.

### 2.2. Interaction-Oriented Semantic Perception

Semantic perception provides the object-level information required to establish correspondence between physical manufacturing components and their virtual representations. In the proposed framework, visual perception serves not only to recognize object categories but also to provide coarse image-space localization for subsequent three-dimensional spatial analysis.

For manufacturing scene reconstruction, the perception module should balance recognition performance and computational efficiency. A computationally expensive detector may increase processing latency and constrain the responsiveness of subsequent virtual scene reconstruction. Therefore, an interaction-oriented lightweight object detector is employed to provide object identity information and approximate image-space locations with limited computational overhead.

Specifically, the visual detector processes the RGB image and generates object categories together with their corresponding two-dimensional bounding boxes. The predicted category provides the semantic key for retrieving the corresponding digital asset from the predefined CAD library. Meanwhile, the bounding box defines an image-space region of interest associated with the detected physical object. This region is used to constrain the subsequent depth-image and point-cloud processing, reducing the amount of irrelevant spatial information that needs to be analyzed.

The proposed perception strategy adopts a coarse-to-fine organization. The lightweight detector first identifies the object and determines its approximate image-space location [12,13]. The corresponding depth information is then used to obtain object-level geometric data, from which more precise three-dimensional position and orientation can be estimated. This division of responsibilities allows the visual detector to focus on efficient semantic identification, while detailed spatial estimation is performed using depth-based geometric information.

Through this process, semantic perception establishes the initial association between physical objects and their potential virtual counterparts. Its outputs also provide spatial constraints for subsequent point-cloud analysis, forming the visual interface between physical scene observation and semantic–spatial mapping.

### 2.3. Spatial State Perception

Object categories and two-dimensional bounding boxes are insufficient to fully describe the physical configuration required for virtual scene reconstruction. To align a virtual model with its physical counterpart, the spatial state of each object must include both its three-dimensional position and orientation. This is particularly relevant for asymmetric manufacturing components, for which objects with similar positions may have different orientations and therefore require different virtual alignments.

The proposed framework adopts a detection-guided three-dimensional perception process. First, the two-dimensional bounding box generated by semantic perception is mapped to the corresponding depth image to define a region of interest [14,15,16]. The depth measurements within this region are transformed into three-dimensional coordinates using the intrinsic parameters of the depth sensor, producing a candidate point cloud associated with the detected object.

For an image point (*u*, *v*) with depth value *d*, its three-dimensional coordinates can be expressed as

(1) $$X=\frac{(u-c_{x})d}{f_{x}},Y=\frac{(v-c_{y})d}{f_{y}},Z=d$$

where *f_x_* and *f_y_* denote the focal lengths of the depth camera, and *c_x_* and *c_y_* represent the coordinates of the principal point.

The candidate point cloud may contain background surfaces, neighboring components, or depth noise because the two-dimensional detection region does not necessarily coincide exactly with the physical object boundary. Therefore, object-level point-cloud segmentation is performed to isolate the target component from its surrounding environment. The semantic detection result constrains the region in which geometric processing is conducted, helping reduce irrelevant points while maintaining the association between the point cloud and its detected object category.

After segmentation, the three-dimensional position of the object is estimated from the spatial distribution of the remaining points. The centroid of the segmented point cloud is calculated according to

(2) $$p_{c}=\frac{1}{N}\sum_{i=1}^{N}p_{i}$$

where *N* denotes the number of segmented points and *p_i_* represents the three-dimensional coordinate of the *i*-th point. The resulting centroid provides an estimate of the object’s translational position.

In addition to position, object orientation is estimated from the geometric distribution of the segmented point cloud. The covariance matrix is constructed as

(3) $$C=\frac{1}{N}\sum_{i=1}^{N}\left(p_{i}-p_{c}\right){\left(p_{i}-p_{c}\right)}^{T}$$

Eigenvalue decomposition is used to obtain the principal geometric directions of the point cloud. These directions describe the dominant spatial structure of the observed component and are used to establish an object-oriented coordinate frame. An oriented bounding box (OBB) is then constructed from the principal directions to represent the object’s spatial extent and orientation.

Compared with a centroid-only representation, the combination of centroid-based position estimation and OBB-based orientation estimation provides a more complete spatial description for virtual model alignment. The resulting spatial state includes the three-dimensional position and orientation of the physical component, which can be used to determine the placement and pose of its corresponding CAD model.

Overall, the spatial perception process follows a coarse-to-fine strategy: semantic detection defines the region of interest, depth information generates the candidate point cloud, and point-cloud segmentation and geometric analysis estimate the object’s spatial state. This organization allows semantic and geometric information to complement each other while avoiding the need for the lightweight detector to directly recover detailed three-dimensional geometry.

### 2.4. Digital Asset Association

The final stage of the proposed framework establishes correspondence between the perceived physical objects and their virtual representations. Instead of manually selecting and positioning digital models, the proposed approach uses semantic identification and spatial state estimation to support digital asset retrieval and virtual alignment.

The object category obtained from semantic perception is used as an index to retrieve the corresponding model from a predefined digital asset library. Since standardized manufacturing components commonly have existing CAD representations, the retrieved models can retain their detailed geometric information without requiring complete geometry reconstruction from sensor data [17,18].

After asset retrieval, the estimated spatial state is used to align the virtual model with the corresponding physical object. The three-dimensional position estimated from the segmented point cloud determines the translational component of the virtual object’s placement, while the estimated principal directions or OBB provide the orientation information required for pose alignment. The physical-to-virtual transformation can be represented by the homogeneous transformation matrix:

(4) $$T=\left[\begin{array}{cc} R & t\\ 0^{T} & 1 \end{array}\right]$$

where *R* denotes the estimated rotation matrix and *t* denotes the three-dimensional translation vector.

The retrieved CAD model is instantiated in the Unity environment and aligned according to the estimated spatial state [19]. In this process, semantic information determines which digital asset is selected, while geometric information determines where and how the asset is placed. This separation allows the framework to reuse predefined geometric resources while deriving virtual object configurations from physical scene observations.

The complete mapping pathway consists of object identification, detection-guided point-cloud extraction, spatial state estimation, digital asset retrieval, and virtual model alignment. The resulting virtual scene represents the observed object categories and their spatial arrangement. When physical object configurations change, newly perceived semantic and spatial information can be used to update the corresponding virtual object representations, provided that the relevant observations and mapping results are available.

The proposed semantic–spatial mapping framework thus establishes a connection between physical scene perception and virtual scene reconstruction. Rather than performing open-ended geometric reconstruction, it uses object identity to retrieve existing CAD assets and spatial state estimation to align them, providing a practical basis for scene reconstruction and updating in manufacturing digital twins.

## 3. Interaction-Oriented SF-Lite-YOLOv8n Semantic Perception Module

### 3.1. Motivation and Design Principle

In the proposed physical-scene-driven interaction paradigm, semantic perception provides the essential link between physical manufacturing objects and their corresponding digital representations. The role of the perception module extends beyond conventional object detection, as its output directly determines the selection of digital assets within the virtual environment.

Unlike traditional industrial vision tasks that mainly focus on recognition accuracy or defect identification, digital-twin interaction requires continuous semantic acquisition with low latency. The perception system must rapidly identify physical objects while maintaining sufficient representation capability for distinguishing components with similar structures and appearances.

YOLOv8n provides a suitable foundation for this scenario due to its lightweight architecture and efficient inference capability [20,21,22]. However, directly applying the original YOLOv8n may limit feature representation when dealing with manufacturing components containing complex geometric patterns or subtle inter-class differences. Furthermore, digital-twin interaction requires a better balance between semantic representation capability and computational efficiency.

Therefore, we develop SF-Lite-YOLOv8n, an interaction-oriented lightweight semantic perception network based on YOLOv8n. The proposed network enhances feature representation and prediction efficiency while preserving the lightweight characteristics required for responsive physical–virtual interaction. Its primary objective is to provide reliable object identity information and coarse image-space localization for subsequent spatial-state estimation and digital asset association.

### 3.2. Architecture of SF-Lite-YOLOv8n

SF-Lite-YOLOv8n is developed by optimizing the original YOLOv8n architecture according to the requirements of physical–virtual interaction. The design principle is to enhance semantic representation capability while maintaining efficient inference performance.

The original YOLOv8n adopts a lightweight backbone and feature aggregation strategy, providing an effective balance between detection accuracy and computational cost. However, manufactured objects often exhibit subtle structural variations, complex geometric patterns, and highly similar visual appearances. These characteristics impose higher requirements on discriminative feature extraction for reliable object-level semantic association.

**Figure 2:**
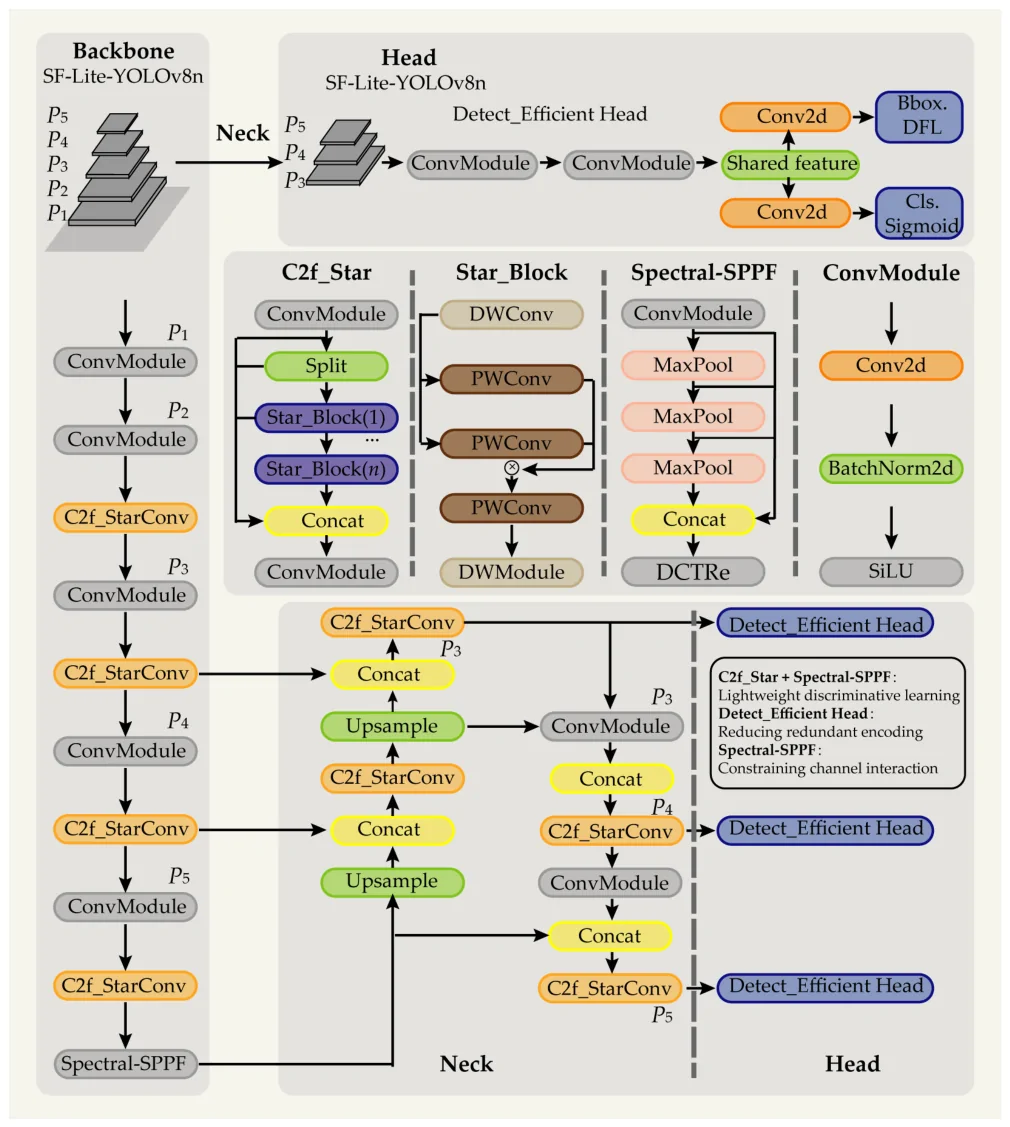
Overall architecture of SF-Lite-YOLOv8n for semantic perception and physical–virtual object association.

To address these challenges, SF-Lite-YOLOv8n introduces targeted improvements in feature extraction, multi-scale feature representation, and prediction efficiency. The overall architecture is illustrated in Figure 2, which consists of three major components: an enhanced feature extraction module based on C2f_Star, a spectral-aware feature aggregation module based on Spectral-SPPF, and an optimized detection head for efficient semantic prediction.

The original multi-scale feature hierarchy is preserved to maintain adaptability to objects with different scales and appearances in manufacturing environments. The proposed C2f_Star module improves discriminative feature learning by enhancing local structural representation, while Spectral-SPPF introduces DCT-based structured feature aggregation to capture complementary feature patterns. Furthermore, the optimized detection head reduces redundant computation while maintaining effective semantic prediction capability.

Through these improvements, SF-Lite-YOLOv8n provides a lightweight semantic perception interface for physical-object-driven digital-twin scene reconstruction. Its outputs are used to establish semantic links between physical manufacturing objects and their corresponding virtual assets, while the associated image-space regions guide subsequent point-cloud processing.

### 3.3. Feature Representation Enhancement Through C2f_Star

The visual distinction among electrical components often relies on subtle structural patterns, local geometric characteristics, and fine-grained appearance cues. Therefore, effective feature extraction requires sufficient capability to capture discriminative information while maintaining a compact computational footprint.

Increasing network depth or introducing complex feature interaction mechanisms can improve representation capability, but often leads to increased computational cost and memory consumption [22,23]. To achieve an improved balance between feature discrimination and efficiency, SF-Lite-YOLOv8n introduces the C2f_Star module by redesigning the feature aggregation process of the original C2f structure.

**Figure 3:**
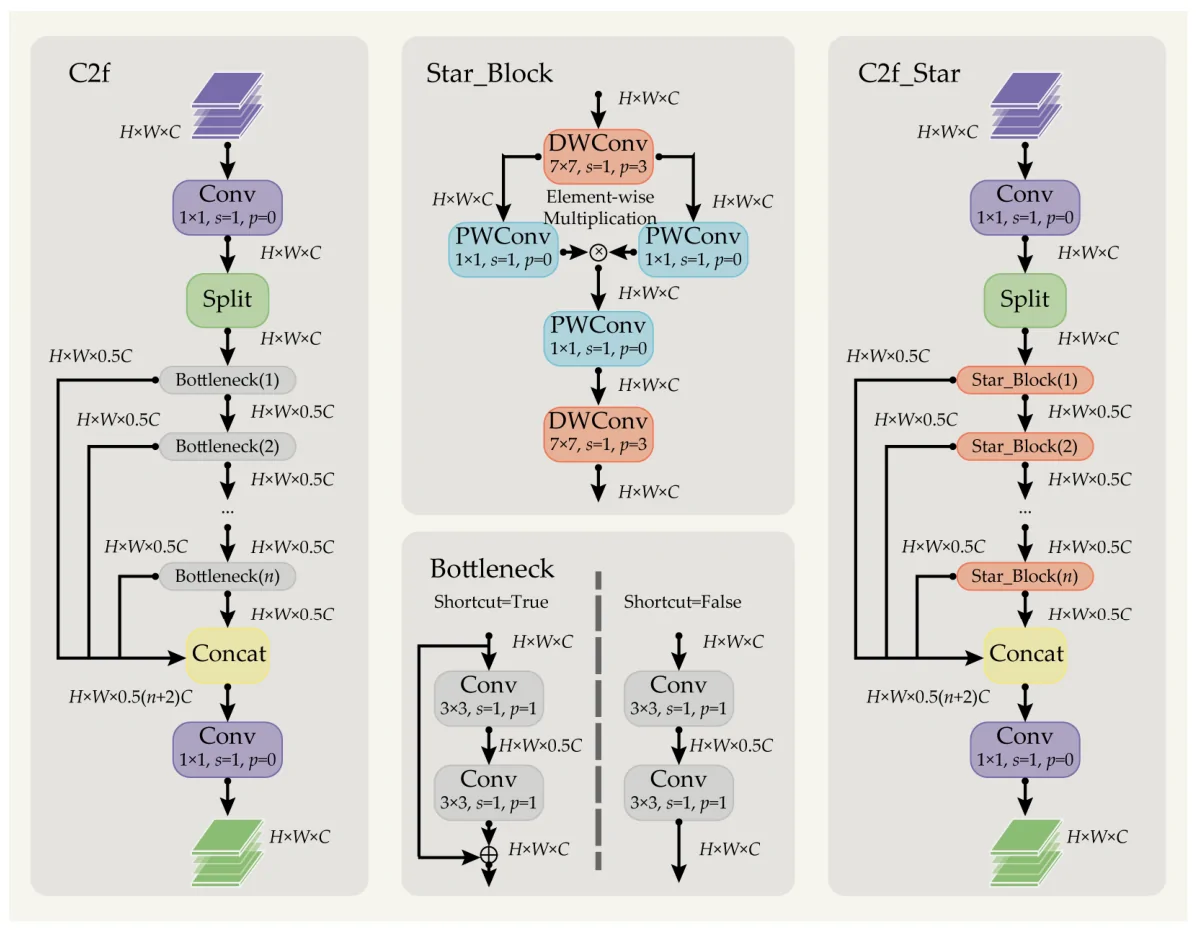
Structure comparison between the original C2f module and the proposed C2f_Star module.

The proposed C2f_Star retains the cross-stage partial feature aggregation strategy of C2f while incorporating a lightweight Star-based interaction block for efficient feature modulation. Instead of relying on computationally expensive dependency modeling, the proposed design enhances local feature interaction through spatial modeling and multiplicative feature coupling. The detailed structure of C2f_Star is illustrated in Figure 3.

Specifically, the integrated Star_Block first performs local spatial modeling via a depthwise convolution:

*z* = *DWConv*_7×7_(*x*) (5)

where *DWConv*(‧) denotes depthwise convolution, which captures spatial patterns independently across channels with substantially fewer parameters compared with standard convolution.

Subsequently, two parallel 1 × 1 pointwise projection branches, parameterized by *W*_1_ and *W*_2_, are applied to generate complementary feature representations:

*g*_1_ = *W*_1_*z*, *g*_2_ = *W*_2_*z* (6)

The generated feature representations are combined through multiplicative interaction:

*g* = *ReLU*6(*W*_1_*z*)⊙(*W*_2_*z*) (7)

where ⊙ denotes element-wise multiplication. This gated interaction enables adaptive feature selection by amplifying informative responses while suppressing irrelevant variations, without requiring additional attention matrices.

The gated feature representation is further refined through a 1 × 1 pointwise projection *W*_3_, a second 7 × 7 depthwise convolution, and a residual connection with stochastic depth regularization:

*y* = *x* + *DropPath*(*DWConv*_7×7_(*W*_3_*g*)) (8)

where *DropPath*(‧) improves feature robustness by introducing stochastic depth during training.

By integrating Star-based feature interaction into the C2f framework, C2f_Star improves the ability of lightweight networks to encode fine-grained structural information while maintaining low computational overhead. In the proposed physical–virtual mapping framework, this enhanced feature representation is intended to support the discrimination of visually similar manufacturing components, thereby providing more reliable semantic information for subsequent digital asset retrieval.

### 3.4. Spectral-Spatial Feature Aggregation Through Spectral-SPPF

Multi-scale feature aggregation plays a crucial role in object detection by integrating local spatial details with large-scale contextual information. The Spatial Pyramid Pooling Fast (SPPF) module expands the receptive field through sequential pooling operations and provides an efficient solution for multi-scale context extraction [24,25]. However, after multi-scale pooling and channel concatenation, the feature recomposition process in conventional SPPF relies on a fully learnable dense channel transformation. Specifically, each output channel is generated through interactions with all concatenated feature channels, resulting in a large number of independently optimized parameters. Such unconstrained channel mixing introduces redundant parameterization and increases optimization complexity, which is undesirable for lightweight detectors deployed in resource-constrained environments.

To address this limitation, we propose Spectral-SPPF, a structured feature recomposition mechanism that introduces a fixed DCT-based prior into the channel reconstruction process. Instead of directly learning an unconstrained dense transformation matrix, Spectral-SPPF first projects the aggregated multi-scale features onto a predefined Discrete Cosine Transform (DCT) basis, generating a structured DCT representation of the feature responses [26,27]. Subsequently, a lightweight learnable reconstruction layer is employed to adaptively combine the retained DCT components for task-specific feature refinement. By separating fixed feature representation from learnable feature adaptation, the proposed method constrains the feature reconstruction space while maintaining sufficient flexibility for object detection.

**Figure 4:**
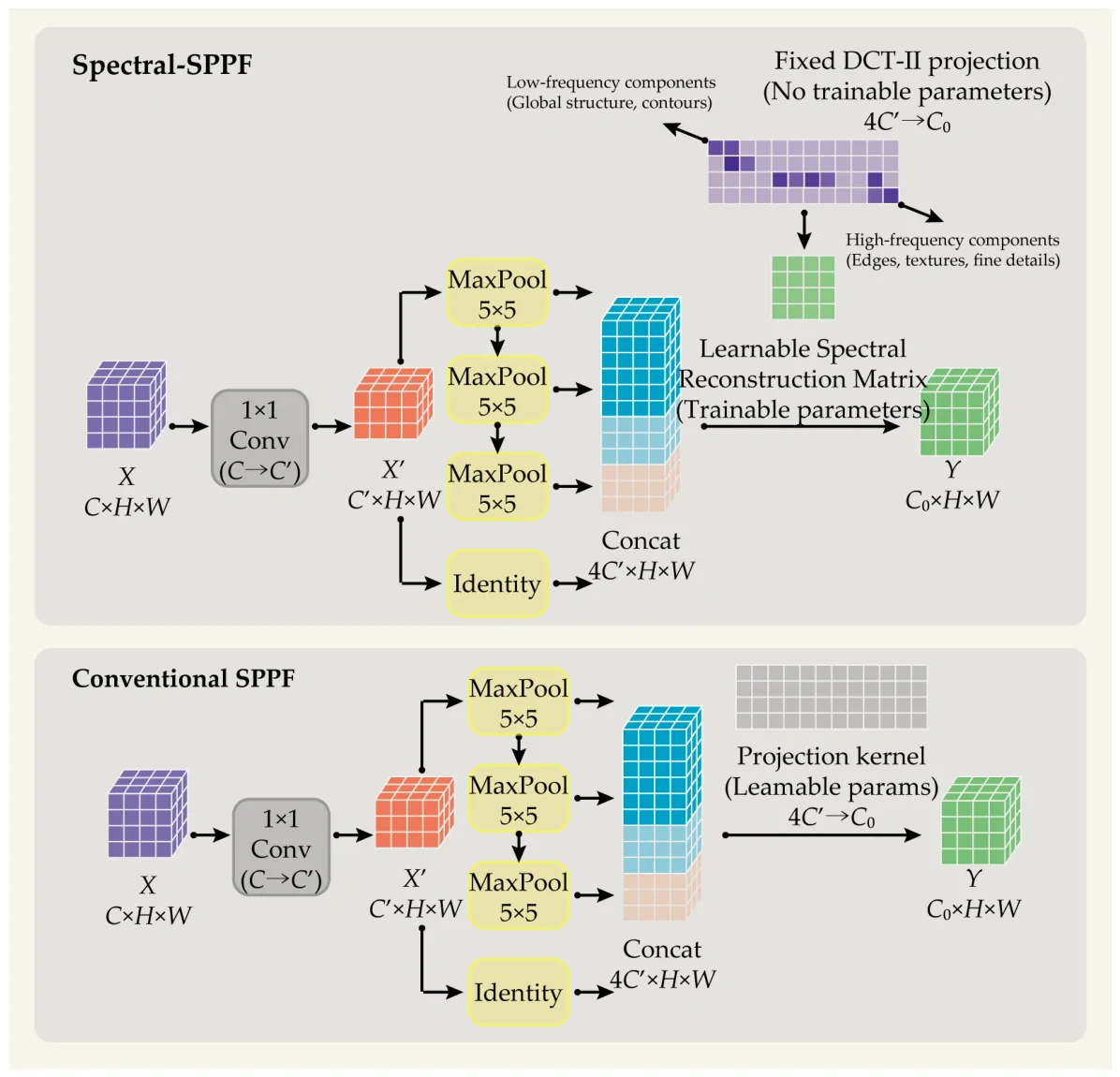
Comparison of SPPF and Spectral-SPPF for multi-scale feature aggregation.

The overall architecture of the proposed Spectral-SPPF module is illustrated in Figure 4. Given an input feature map *X* ∈ ℝ*^C^*^×*H*×*W*^, the feature representation is first transformed through a lightweight channel adaptation layer to reduce redundant channel dimensions while preserving discriminative information. Specifically, a 1 × 1 convolution is employed to obtain a compact feature representation:

*U* = *δ*(*W_i_*∗*X*) (9)

where *W_i_* denotes the learnable channel projection kernel and *δ*(‧) represents the nonlinear activation function. The resulting feature map is subsequently processed by sequential max-pooling operations to capture multi-scale contextual information:

*P*_1_ = Pool_5×5_(*U*), *P*_2_ = Pool_5×5_(*P*_1_), *P*_3_ = Pool_5×5_(*P*_2_) (10)

where the three pooling branches correspond to effective receptive fields of 5 × 5, 9 × 9, and 13 × 13, respectively. The resulting multi-scale representations are concatenated along the channel dimension:

*Z* = Concat(*U*, *P*_1_, *P*_2_, *P*_3_) (11)

where *Z* ∈ ℝ^4*C*′×*H*×*W*^ denotes the aggregated contextual representation.

In the conventional SPPF module, the concatenated feature is reconstructed using a fully learnable dense channel transformation:

*Y_s_* = *σ*(*W_s_Z*) (12)

where *W_s_* ∈ ℝ*^C^*^0×4*C*′^ is the learnable reconstruction matrix and *σ*(‧) represents the nonlinear activation function.

Since each output channel independently learns interactions with all input channels, the dense transformation inevitably introduces redundant channel correlations and increases the number of trainable parameters.

To overcome this limitation, Spectral-SPPF introduces a fixed spectral constraint into the feature recomposition process. Specifically, the concatenated feature representation is first mapped into a spectral coordinate space using a predefined DCT basis:

*Z_f_* = Φ*Z* (13)

where *D* ∈ ℝ^4*C*′×4*C*′^ denotes the normalized orthonormal DCT-II matrix. When *C*_0_ < 4*C*′, the actual projection matrix is defined as Φ = *P*_*C*_0__*D*, where *P*_*C*_0__ selects the retained DCT coefficients. Therefore, Φ ∈ ℝ^*C*_0_^^×4*C*′^ represents a truncated DCT-II projection rather than a square orthogonal matrix. Different from conventional learnable channel mixing, the DCT projection provides a deterministic structured coordinate transformation for channel-wise feature representation. The retained DCT components are subsequently used for adaptive feature reconstruction.

After spectral projection, a lightweight adaptive reconstruction layer is applied:

*Y* = *σ*(*AZ_f_*) (14)

where *A* ∈ ℝ^*C*_0_^^×*C*_0_^ denotes a learnable spectral reconstruction matrix shared across all spatial locations, which adaptively models the interactions among the retained DCT components. Therefore, the number of trainable parameters in the spectral reconstruction matrix is

*N_A_* = *C*_0_ × *C*_0_ (15)

Compared with the original dense reconstruction, the proposed formulation reduces the learnable reconstruction parameter space from *C*_0_ × 4*C*′ to *C*_0_ × *C*_0_, while the fixed DCT basis constrains the channel reconstruction space without introducing additional trainable parameters.

Compared with direct dense channel mixing, the proposed strategy reformulates feature reconstruction through structured DCT projection and compact adaptive modulation. By constraining feature interactions within a structured representation space, Spectral-SPPF reduces redundant parameterization while preserving adaptive representation capability. In the proposed detection framework, this design provides multi-scale contextual feature aggregation while supporting the computational efficiency required for physical scene perception.

### 3.5. Interaction-Aware and Redundancy-Aware Detection Head

In the proposed physical-scene-driven interaction paradigm, the detection head serves as the semantic and coarse-localization interface between the physical manufacturing scene and the subsequent three-dimensional perception process. Its role therefore differs from that of conventional object detection heads, where the detection output is typically regarded as the final perception result. In the proposed framework, the detection head provides the semantic identity and coarse spatial region required to initiate subsequent point-cloud-based geometric perception.

The category prediction provides the semantic key for retrieving the corresponding digital asset from the predefined model library, while the predicted bounding box defines the image-space region associated with the physical object. This region is subsequently projected onto the depth image to generate a candidate three-dimensional point cloud for object-level segmentation and spatial-state estimation. Consequently, the detection head is not required to directly recover the complete three-dimensional pose of the object. Instead, it provides reliable semantic information and coarse localization at low computational cost, while precise position and orientation are obtained from the downstream point-cloud perception stage.

This division of responsibilities motivates an interaction-oriented redesign of the detection head. Since the subsequent geometric module performs fine-grained spatial estimation, unnecessary feature transformations within the visual detection stage should be minimized to maintain efficient perception and support responsive virtual scene reconstruction.

In conventional YOLOv8, the decoupled detection head independently processes classification and regression branches. Although this design provides task-specific feature representations, the two branches repeatedly encode the same input feature maps, resulting in redundant spatial transformations. Such redundancy becomes particularly significant for high-resolution feature maps and is undesirable in an interaction-oriented system where visual perception is expected to operate efficiently.

**Figure 5:**
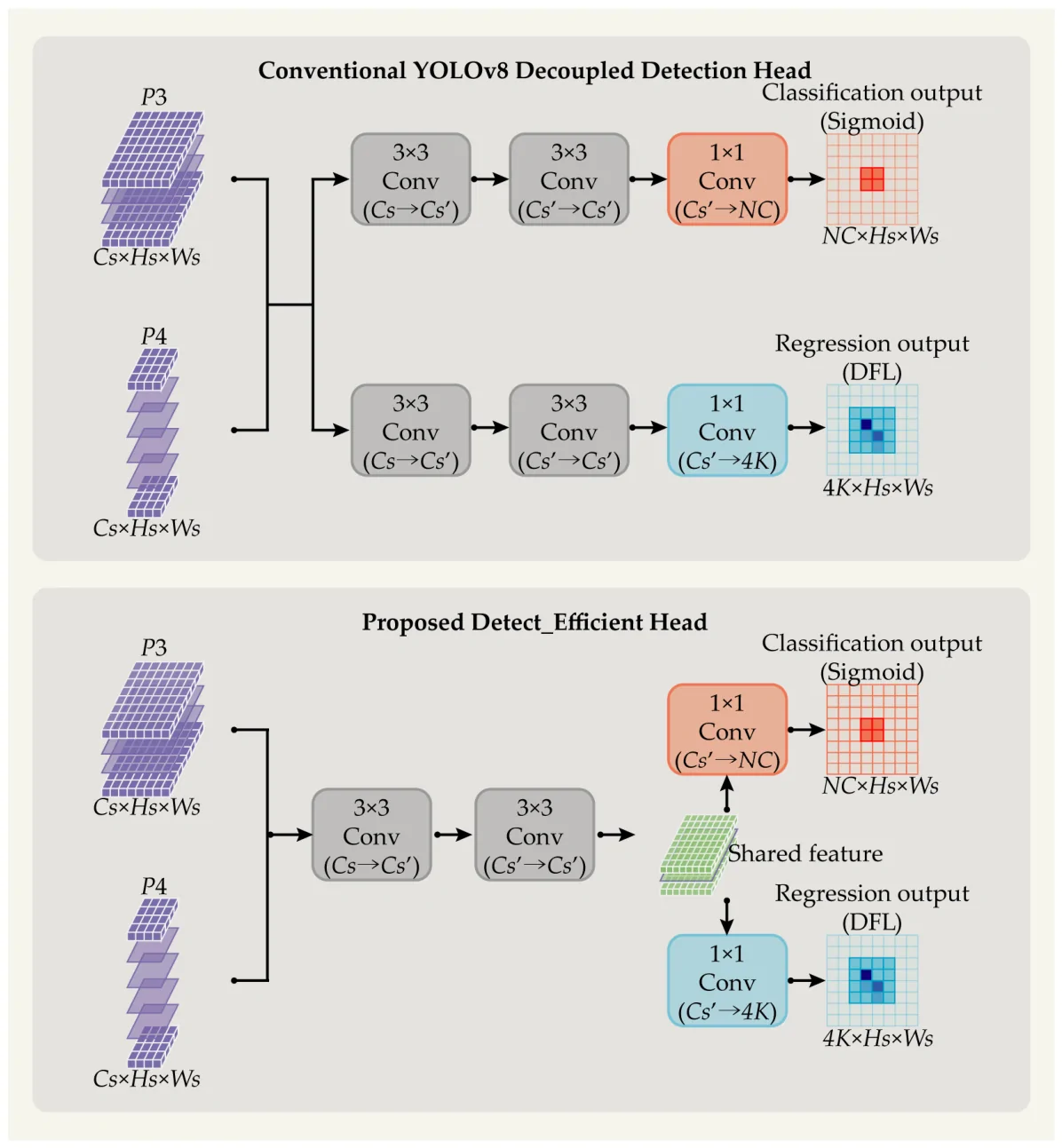
Architecture of the proposed Detect_Efficient head with shared feature encoding and lightweight task-specific projections.

To address this issue, we introduce Detect_Efficient, an interaction-aware and redundancy-aware detection head that reformulates the conventional decoupled architecture as a shared feature-shaping pathway followed by lightweight task-specific projections. The overall design is illustrated in Figure 5. For an input feature representation

(16) $$F_{s}^{\mathrm{shared}}=\Psi_{s}(F_{s})$$

where Ψ(‧) denotes the shared feature-shaping operation, consisting of lightweight convolutional transformations for local spatial aggregation and feature refinement.

Unlike conventional decoupled detection heads, in which classification and regression branches independently perform spatial feature encoding, Detect_Efficient computes this intermediate representation only once. The resulting shared representation is subsequently reused by the two prediction branches.

The regression and classification outputs are then generated through independent lightweight projections:

(17) $$R_{s}=W_{s}^{\mathrm{reg}}*T_{s},\;P_{s}=W_{s}^{\mathrm{cls}}*T_{s}$$

where * denotes a convolution operation, and $W_{s}^{reg}$ and $W_{s}^{cls}$ represent task-specific 1 × 1 projection kernels for bounding box regression and classification, respectively.

This formulation concentrates the computationally expensive spatial feature encoding into a single shared pathway, while retaining independent prediction spaces for the two tasks. The classification branch can therefore preserve category-specific discrimination, whereas the regression branch maintains the localization capability required for generating coarse object regions.

The efficiency improvement of Detect_Efficient originates from two complementary aspects. First, redundant spatial feature encoding between classification and regression branches is reduced by sharing the intermediate representation. Second, branch-specific transformations are simplified into lightweight point-wise projections, reducing unnecessary channel expansion and repeated convolutional operations. As a result, the computational burden of the detection head is reduced while retaining the prediction outputs required by the object detection framework.

The proposed design is also consistent with the hierarchical perception strategy described in Section 2.3. The bounding-box regression output is intentionally used as a coarse localization result rather than as the final spatial state of the physical object. The predicted bounding box identifies the approximate image-space extent of the target and constrains the subsequent depth and point-cloud processing region. Fine-grained three-dimensional localization and orientation estimation are subsequently performed using the segmented object point cloud.

To maintain compatibility with the original YOLOv8 prediction framework, Detect_Efficient preserves the distribution-based regression formulation. Specifically, each bounding box side is modeled as a discrete probability distribution over *k* bins (typically *K* = 16). For a predicted probability vector *p* ∈ ℝ*^K^*, the expected distance is calculated as follows:

(18) $$\hat{d}=\sum_{k=0}^{K-1}k\cdot p_{k}$$

where *p_k_* denotes the predicted probability of the *k*-th distance bin. denotes the predicted probability of the *k*-th distance bin.

The four estimated distances are subsequently decoded to construct the two-dimensional bounding box and transformed into the original image coordinate system according to the corresponding feature stride. The classification branch outputs category logits, which are converted into class probabilities through sigmoid activation.

Because the decoding procedure and output semantics remain unchanged, the proposed head modifies the internal feature encoding pathway without altering the fundamental prediction formulation of YOLOv8. This preserves compatibility with the existing detection framework while reducing redundant computation.

From the perspective of physical–virtual interaction, Detect_Efficient therefore performs two complementary functions. At the semantic level, it generates the object category required for digital asset retrieval. At the spatial level, it provides a computationally efficient coarse bounding box that guides subsequent point-cloud processing. The detection result is consequently no longer treated as an isolated recognition output, but as an intermediate representation connecting visual perception with three-dimensional geometric reasoning.

Through this interaction-aware and redundancy-aware design, SF-Lite-YOLOv8n provides a lightweight visual front end for physical-scene-driven digital-twin interaction. The detector efficiently determines what the physical object is and approximately where it is, while the subsequent point-cloud perception stage determines its precise three-dimensional position and orientation. This hierarchical division of perception tasks reduces unnecessary computational overhead in the detection stage while preserving the spatial information required for accurate physical–virtual correspondence.

### 3.6. Summary of SF-Lite-YOLOv8n

This section presents SF-Lite-YOLOv8n, an interaction-oriented lightweight semantic perception network designed for the physical-scene-driven digital-twin framework.

Unlike conventional detector optimization approaches that mainly pursue higher benchmark performance, SF-Lite-YOLOv8n is developed according to the requirements of physical–virtual interaction. The network improves semantic representation capability through C2f_Star-based feature enhancement, strengthens structural information extraction through Spectral-SPPF-based multi-scale aggregation and optimizes semantic output through an interaction-aware detection head.

Importantly, the original multi-scale feature hierarchy of YOLOv8n is retained. Unlike scale-specific pruning strategies that may depend on the object-size distribution of a particular dataset, the proposed design maintains adaptability across different manufacturing scenarios.

As a semantic interface within the proposed framework, SF-Lite-YOLOv8n provides reliable object identity information for digital asset association. Combined with subsequent spatial state estimation, it enables physical manufacturing objects to directly participate in digital-twin scene construction.

## 4. Experimental Validation of Physical-Scene-Driven Scene Reconstruction

### 4.1. Experimental Setup and Evaluation Protocol

To evaluate the proposed physical-scene-driven semantic–spatial mapping framework, experiments are conducted to examine semantic perception performance, physical–virtual mapping accuracy, computational efficiency, and virtual scene reconstruction. The experimental process comprises three main stages: physical scene acquisition, semantic–spatial understanding, and virtual scene generation.

An RGB-D sensor (RealSense D435; Intel Corporation, Santa Clara, CA, USA) is used to acquire manufacturing scenes containing different industrial components. The RGB images are processed by the semantic perception module to identify object categories and obtain the corresponding image regions. The depth images are aligned with the RGB images and converted into point clouds for three-dimensional spatial analysis. For digital-twin construction and collision verification, a predefined digital asset library of CAD models developed using SOLIDWORKS 2024 (Dassault Systèmes, Vélizy-Villacoublay, France) is used for virtual model retrieval and instantiation.

The proposed framework establishes semantic–spatial correspondence by using detected object categories as retrieval indices for the digital asset library and point-cloud-based geometric information to estimate the spatial states of physical components. The estimated position and orientation are then used to align the retrieved CAD models in the Unity 2022.3 environment (Unity Technologies, San Francisco, CA, USA).

Three experiments are conducted. First, an ablation study is performed on the public MVTec ITODD dataset to evaluate the contributions of the proposed modules in SF-Lite-YOLOv8n. Second, different physical–virtual mapping strategies are compared using a self-collected manufacturing scene dataset consisting of 20 scenes with different component arrangements. Third, a representative manufacturing scene is selected to demonstrate the process of semantic identification, spatial state estimation, CAD asset association, and Unity-based virtual scene reconstruction.

The evaluation metrics include object detection accuracy, model complexity, inference speed, spatial mapping errors, and correspondence latency. Detection performance is assessed using Precision, Recall, and mAP@0.5, while computational efficiency is evaluated in terms of parameter number, GFLOPs, model size, and inference speed. Physical–virtual mapping performance is evaluated using position error, orientation error, and correspondence latency. The latency measurement includes semantic perception, digital asset retrieval, spatial estimation, and virtual model alignment, but excludes Unity rendering and visualization time.

### 4.2. Ablation Study of SF-Lite-YOLOv8n on MVTec ITODD Dataset

An ablation study is conducted on the public MVTec ITODD dataset to evaluate the contribution of each proposed component to object detection performance and computational efficiency. The dataset contains industrial objects with diverse appearances and geometric structures, providing a benchmark for assessing semantic perception in manufacturing scenarios [28].

The original YOLOv8n is used as the baseline model. The proposed modules are progressively incorporated to investigate their individual and combined effects. First, the original C2f module is replaced with C2f_Star to evaluate the influence of the modified feature extraction structure. Spectral-SPPF is then introduced to enhance multi-scale feature representation through spectral-aware feature aggregation. Finally, Detect_Efficient is integrated to form the complete SF-Lite-YOLOv8n architecture.

All configurations are trained and evaluated under the same experimental conditions to ensure comparability. Precision, Recall, mAP@0.5, parameter number, GFLOPs, model size, and inference speed are used as evaluation metrics. The quantitative results are summarized in Table 1.

As shown in Table 1, the baseline YOLOv8n achieves an mAP@0.5 of 0.923 with 3.01 million parameters and 8.1 GFLOPs. After replacing the original C2f module with C2f_Star, the parameter number decreases to 2.80 million, while mAP@0.5 increases to 0.930. This result indicates that the modified feature extraction module improves the representation of industrial objects while reducing the model parameter count.

After the introduction of Spectral-SPPF, mAP@0.5 further increases to 0.938, and Recall reaches 0.930. Although the parameter number and computational cost increase at this stage, the improvement in detection performance indicates that spectral-aware multi-scale feature aggregation contributes additional representation capability for objects with complex geometric characteristics.

With the integration of Detect_Efficient, the complete SF-Lite-YOLOv8n achieves an mAP@0.5 of 0.940, while the parameter number decreases to 1.98 million and computational complexity decreases to 6.2 GFLOPs. The inference speed increases to 83 FPS. Compared with the baseline YOLOv8n, the complete model therefore achieves higher detection accuracy with fewer parameters and lower computational complexity.

### 4.3. Physical–Virtual Mapping Evaluation

To evaluate the accuracy and efficiency of physical–virtual correspondence, experiments are conducted on a self-collected manufacturing scene dataset consisting of 20 scenes from a laboratory-scale production environment. The scenes contain different spatial arrangements of typical industrial components, including metal and plastic connectors, shaft brackets, and nuts. Corresponding CAD models of these components are available in a predefined digital asset library.

An RGB-D sensor is used to capture each manufacturing scene. RGB images provide semantic information for object identification, while aligned depth information is converted into point clouds for three-dimensional spatial estimation. The spatial state of each component is represented by its three-dimensional position and orientation. The position is estimated from the centroid of the segmented point cloud, and the orientation is obtained through OBB fitting based on the geometric distribution of the object points.

Three physical–virtual mapping strategies are compared.

(1) YOLOv8n-seg

YOLOv8n-seg is adopted as a vision-based mapping strategy [29]. The model obtains object categories and segmentation masks from RGB images, and the detected categories are used to retrieve the corresponding digital assets. The object regions are derived from image segmentation, and the resulting image-space information is used to support virtual object placement.

(2) RF-DETR + Point Cloud

In this strategy, RF-DETR is used for object detection, while point-cloud processing is employed for three-dimensional spatial estimation. The detected object regions are associated with the corresponding depth information to obtain geometric representations of the physical components. The resulting spatial information is then used to align the retrieved CAD models in the virtual environment [30].

(3) SF-Lite-YOLOv8n + Point Cloud

The proposed strategy integrates semantic perception and spatial state estimation. SF-Lite-YOLOv8n provides object identity information for digital asset retrieval, while the point cloud provides geometric constraints for determining object position and orientation. The retrieved CAD models are aligned according to the estimated spatial states and instantiated in the virtual environment.

The evaluation focuses on the accuracy and efficiency of physical–virtual correspondence. The quantitative metrics include position error, orientation deviation, and correspondence latency.

The position error is calculated as the Euclidean distance between the reconstructed object position and the reference position:

(19) $$E_{p}=\sqrt{{\left(x_{r}-x_{v}\right)}^{2}+{\left(y_{r}-y_{v}\right)}^{2}+{\left(z_{r}-z_{v}\right)}^{2}}$$

where (*x_r_*, *y_r_*, *z_r_*) and (*x_v_*, *y_v_*, *z_v_*) represent the reference and reconstructed positions, respectively.

The orientation error is represented by the equivalent rotation angle between the reference rotation matrix and reconstructed rotation matrix:

(20) $$E_{R}=\cos^{-1}\frac{T_{r}(R_{r}^{T}R_{v})-1}{2}$$

where *R_r_* and *R_v_* denote the reference and reconstructed orientations.

Latency represents the time required to complete one physical–virtual correspondence process, including semantic perception, digital asset retrieval, spatial estimation, and virtual model alignment. It does not include the rendering or visualization time in Unity. All experiments are conducted on a workstation equipped with an NVIDIA GeForce RTX 3060 GPU (12 GB VRAM), an Intel Core i7-8700 CPU, and 32 GB RAM. RGB images are resized to 640 × 640 pixels, and depth images are aligned with the RGB images at the same resolution.

The comparison results across 20 manufacturing scenes are summarized in Table 2. The YOLOv8n-seg strategy achieves an average position error of 9.1 mm and an average orientation error of 7.2°, with an average latency of 85 ms. The correspondence process is established mainly through RGB image-based semantic segmentation and object region extraction. The RF-DETR + Point Cloud strategy achieves an average position error of 3.5 mm and an average orientation error of 2.7°. The average latency reaches 320 ms. The detected object regions are further combined with depth information for three-dimensional spatial estimation. The SF-Lite-YOLOv8n + Point Cloud strategy obtains average position and orientation errors of 2.7 mm and 1.9°, respectively. The average latency is 146 ms. The experimental measurements are obtained from 20 manufacturing scenes containing different arrangements of metal and plastic components.

### 4.4. Case Study of Physical-Scene-Driven Virtual Scene Reconstruction

**Figure 6:**
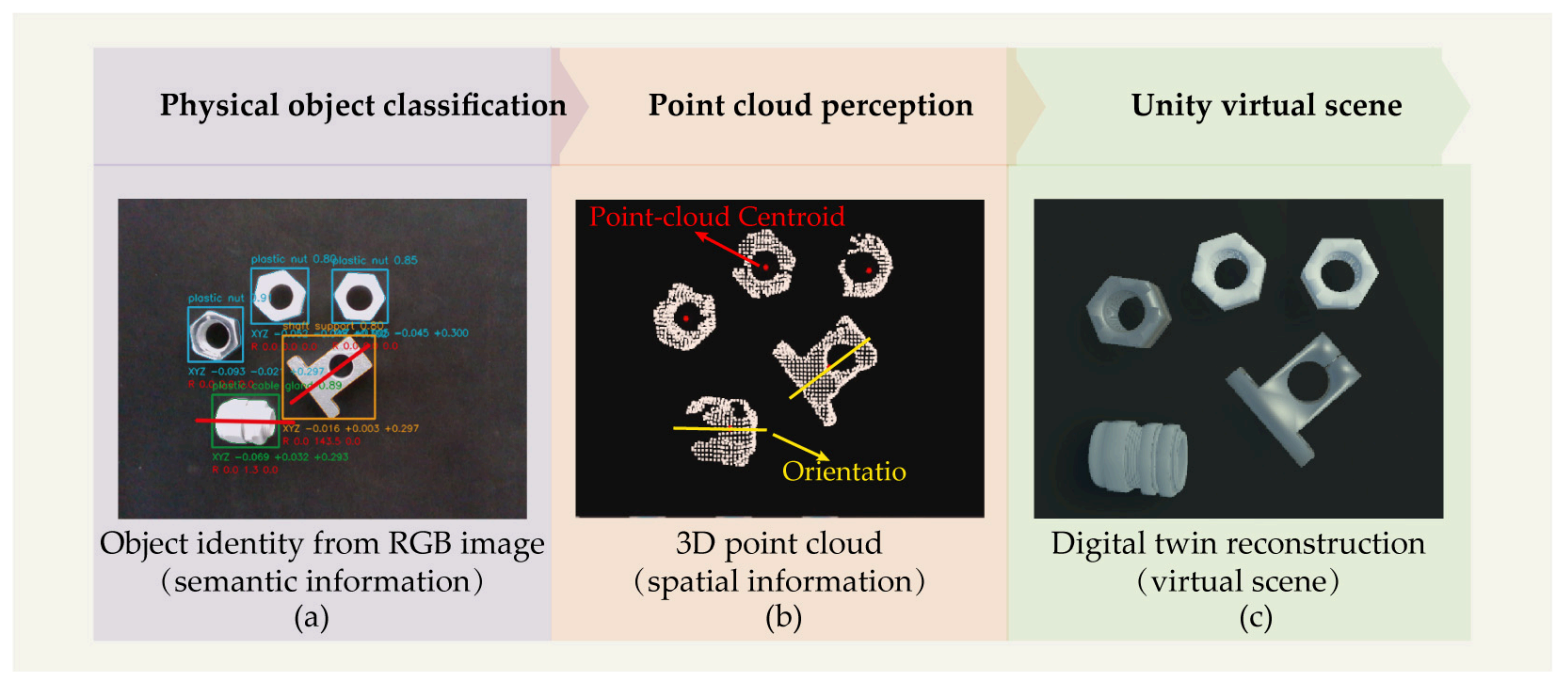
Example of physical-scene-driven digital twin reconstruction from semantic perception to virtual scene generation. (**a**) RGB image with semantic object identification. (**b**) Object-level point cloud extraction and spatial state estimation. (**c**) Unity-based digital twin reconstruction using retrieved CAD assets.

A representative manufacturing scene is selected to demonstrate the physical-to-virtual scene reconstruction process. As illustrated in Figure 6, the process comprises semantic perception, geometric state estimation, digital asset association, and virtual model instantiation.

First, the physical manufacturing scene is captured using an RGB-D sensor. The RGB image is processed by SF-Lite-YOLOv8n to obtain the categories and corresponding image regions of the manufacturing components. The detected semantic information is used to identify the physical objects and retrieve their corresponding CAD models from the predefined digital asset library.

Second, the depth information aligned with the RGB image is converted into a point cloud. The image regions obtained from semantic perception are used to constrain object-level point-cloud extraction. The segmented point clouds are then processed to estimate the three-dimensional position and orientation of each physical component. These spatial states provide the geometric information required for virtual model alignment.

Finally, the retrieved CAD models are imported into the Unity environment and positioned according to the estimated spatial states. The resulting virtual scene represents the identified component categories and their relative spatial arrangement in the observed physical scene. As shown in Figure 6, the reconstructed scene is generated by associating semantic object identification with point-cloud-based spatial estimation and CAD model alignment.

This case study illustrates how the proposed framework connects physical scene observation with virtual scene reconstruction through semantic–spatial correspondence. The use of predefined CAD assets preserves the geometric detail of the virtual components, while point-cloud-based spatial estimation provides the position and orientation information required for alignment. The demonstrated workflow provides a basis for reconstructing virtual scenes from observed manufacturing configurations and for updating virtual object representations when changes in physical states are detected.

## 5. Discussion

The central contribution of this study is a physical-scene-driven semantic–spatial mapping framework that connects physical manufacturing objects with their virtual counterparts to support scene reconstruction and updating in manufacturing digital twins. Rather than relying exclusively on manually configured virtual scenes, the proposed framework uses physical objects as sources of semantic and spatial information. Semantic identity determines which digital asset should be retrieved, while spatial state determines where and how the asset should be represented in the virtual environment. This separation is important because manufacturing components often have predefined CAD models available in digital asset libraries. In such cases, the reconstruction task does not require generating complete object geometry from scratch, but instead focuses on establishing reliable semantic–spatial correspondence between observed objects and existing digital resources. When new physical-scene observations become available, the corresponding semantic and spatial information can also provide the basis for updating the virtual scene.

The experimental results demonstrate the potential of the proposed lightweight semantic perception module for supporting this process. SF-Lite-YOLOv8n achieves an mAP@0.5 of 0.940 on the MVTec ITODD dataset, with 1.98 million parameters and 6.2 GFLOPs, at a measured inference speed of 83 FPS. Relative to the original YOLOv8n baseline, the complete model improves mAP@0.5 from 0.923 to 0.940 while reducing the parameter count from 3.01 million to 1.98 million and computational complexity from 8.1 to 6.2 GFLOPs. The measured inference speed also increases from 76 to 83 FPS. These results indicate that the proposed modifications improve the balance between detection performance and computational efficiency. In the proposed framework, the detector primarily provides semantic identity and image-space localization for downstream spatial processing, rather than serving as the source of complete three-dimensional object geometry. This task allocation allows the perception module to focus on the information needed for digital asset association while leaving precise spatial estimation to point-cloud processing.

The ablation results provide further insight into the contributions of the individual network components. Replacing the original C2f module with C2f_Star increases mAP@0.5 from 0.923 to 0.930 while reducing the parameter count from 3.01 million to 2.80 million. Introducing Spectral-SPPF further increases mAP@0.5 to 0.938 and recall to 0.930, although the associated parameter count and computational cost also increase. The subsequent integration of Detect_Efficient reduces the model from 3.18 million to 1.98 million parameters and from 8.4 to 6.2 GFLOPs, while increasing the measured inference speed from 77 to 83 FPS. Together, these results suggest that the efficiency of the final network is associated not only with the reduction in individual operations, but also with the organization of feature representation and prediction within the network. The combination of feature enhancement, multi-scale aggregation, and an efficient detection head provides a compact semantic perception module for the subsequent physical–virtual mapping process.

The mapping evaluation further illustrates the role of integrating semantic perception with three-dimensional spatial estimation. Across the 20 self-collected manufacturing scenes, the YOLOv8n-seg strategy achieves a mean position error of 9.1 mm and a mean orientation error of 7.2°, with an average latency of 85 ms. The RF-DETR combined with point-cloud processing achieves mean position and orientation errors of 3.5 mm and 2.7°, respectively, with an average latency of 320 ms. The proposed SF-Lite-YOLOv8n combined with point-cloud processing achieves mean position and orientation errors of 2.7 mm and 1.9°, respectively, with an average latency of 146 ms. These results show that incorporating point-cloud-based spatial information is associated with improved spatial mapping accuracy compared with the evaluated image-based strategy. The proposed combination also achieves lower measured latency than the RF-DETR-based point-cloud strategy under the reported experimental setup. The results therefore support the use of lightweight semantic perception coupled with geometric processing for establishing physical–virtual correspondence in the evaluated manufacturing scenes.

An important aspect of the proposed framework is the complementary role of semantic and spatial information. Object categories provide the retrieval keys for selecting predefined CAD assets, whereas point-cloud geometry provides the spatial constraints for estimating object positions and orientations. The semantic and geometric stages therefore address different aspects of the mapping problem. This division avoids relying on image-based recognition alone to determine three-dimensional object states and allows the virtual representation to retain the geometric characteristics of the corresponding CAD assets. The Unity-based case study illustrates how the outputs of object detection and point-cloud processing can be integrated to associate physical objects with virtual models and reproduce their observed spatial arrangement. This association also provides a basis for updating virtual object configurations when new physical-scene observations indicate changes in object states.

For dynamic manufacturing environments, the usefulness of this framework lies in the potential to reuse the semantic–spatial mapping process as the physical scene evolves. Changes in object position or orientation can, in principle, be reflected by updating the spatial states of their associated virtual objects. Similarly, changes in the set of detected objects can provide information for revising the virtual scene composition. However, the performance of such updates depends on the reliability of object detection, point-cloud segmentation, spatial-state estimation, and the association of observations with existing virtual objects. In particular, the current mapping evaluation is based on 20 manufacturing scenes with different object arrangements. These results characterize mapping accuracy and processing latency under the evaluated conditions, but do not independently establish the performance of continuous object tracking or end-to-end synchronization during uninterrupted manufacturing operation. Further evaluation with sequential observations and controlled physical-scene changes would be required to quantify update frequency, response delay, and scene consistency over time.

Several limitations should nevertheless be considered. First, the semantic perception experiments are based on MVTec ITODD, while the physical–virtual mapping evaluation uses 20 self-collected manufacturing scenes. The evaluated objects and scene configurations therefore represent a limited subset of manufacturing environments. The current results do not establish generalization to unseen object categories, substantially different illumination conditions, severe occlusion, highly dynamic scenes, or large domain shifts. Second, the mapping framework assumes that corresponding CAD assets are available in a predefined digital library. Consequently, the method focuses on semantic asset association and spatial alignment rather than open-ended geometric reconstruction. This assumption is suitable for standardized manufacturing components but limits direct application to previously unseen objects for which no corresponding digital asset exists. In addition, spatial-state estimation based on point-cloud geometry may be affected by incomplete observations, occlusion, measurement noise, and geometric symmetry. These factors may introduce uncertainty in object position and orientation and consequently affect the alignment of virtual models.

A further limitation concerns practical deployment and responsiveness. The measured latency includes semantic perception, digital-asset retrieval, spatial estimation, and virtual-model alignment, but excludes Unity rendering and visualization time. The reported average latency of 146 ms should therefore be interpreted as the correspondence-processing latency of the proposed pipeline rather than the complete end-to-end delay between a physical change and its display in the virtual environment. Moreover, parameter count and GFLOPs alone do not fully determine execution speed on a particular edge platform. Memory access, operator implementation, hardware architecture, and rendering overhead can influence practical throughput. Future work should therefore evaluate the framework on representative industrial edge hardware, investigate hardware-aware optimization, and measure the complete physical-to-virtual update delay under continuous operation.

Overall, the findings indicate that physical-scene-driven semantic–spatial mapping provides a practical basis for connecting physical manufacturing objects with their virtual representations. By integrating object identity, spatial-state estimation, and predefined CAD assets within a unified pipeline, the proposed framework supports the reconstruction of virtual manufacturing scenes from physical observations. Its extension to continuously updated scenes depends on reliable repeated perception and spatial estimation, as well as efficient updating of the associated virtual objects. These findings highlight the importance of designing perception and mapping methods around the semantic and geometric information required for physical–virtual correspondence, rather than optimizing visual recognition in isolation.

## 6. Conclusions

This study presents a physical-scene-driven semantic–spatial mapping framework to support scene reconstruction and updating in manufacturing digital-twin systems. The proposed framework connects physical manufacturing objects with their virtual counterparts by integrating lightweight visual perception, point-cloud-based spatial-state estimation, digital asset retrieval, and Unity-based virtual representation. Semantic information obtained from object detection is used to retrieve corresponding CAD assets from a predefined library, while point-cloud processing estimates the three-dimensional positions and orientations required for virtual model alignment. This approach provides a physical-to-virtual mapping pathway that reduces dependence on manual scene configuration and supports the reconstruction of virtual scenes based on observed manufacturing conditions.

To provide an efficient semantic perception module, SF-Lite-YOLOv8n was developed by integrating C2f_Star, Spectral-SPPF, and Detect_Efficient. On the MVTec ITODD dataset, the proposed detector achieves an mAP@0.5 of 0.940 with 1.98 million parameters and 6.2 GFLOPs, at a measured inference speed of 83 FPS. In the physical–virtual mapping evaluation across 20 self-collected manufacturing scenes, the integrated SF-Lite-YOLOv8n and point-cloud pipeline achieves mean position and orientation errors of 2.7 mm and 1.9°, respectively, with an average correspondence-processing latency of 146 ms, excluding Unity rendering. These results demonstrate the feasibility of combining lightweight semantic perception with geometric point-cloud analysis to establish accurate and computationally efficient physical–virtual correspondence under the evaluated conditions.

The broader contribution of this work is the development of a physical-scene-driven approach in which semantic identity and spatial state jointly determine the association and alignment of virtual objects. By linking observed physical objects to predefined digital assets, the framework provides a basis for reconstructing virtual manufacturing scenes and updating object configurations according to newly perceived physical states, without requiring complete geometric reconstruction of each object. The current evaluation establishes the semantic perception and spatial mapping capabilities of the framework, while continuous scene updating and end-to-end synchronization under changing manufacturing conditions remain important aspects for further validation.

Future work will focus on extending the framework towards continuous scene reconstruction under dynamic manufacturing conditions, including the reliable identification of object-state changes, persistent association of physical objects with virtual counterparts, and timely updating of virtual scenes. Further investigation of unseen components, severe occlusion, larger domain shifts, and deployment on industrial edge hardware will also be necessary to assess the framework’s robustness and practical applicability in real manufacturing environments.

## Figures and Tables

**Table 1:** Ablation study of SF-Lite-YOLOv8n on the MVTec ITODD dataset.

Configuration	Parameters (M)	GFLOPs	Precision	Recall	mAP@0.5	Model Size (MB)	FPS
Baseline ^1^	3.01	8.1	0.928	0.914	0.923	6.0	76
+C2f_Star ^2^	2.80	7.6	0.934	0.921	0.930	5.6	79
+C2f_Star +Spectral-SPPF ^3^	3.18	8.4	0.941	0.930	0.938	6.3	77
SF-Lite-YOLOv8n ^4^	1.98	6.2	0.941	0.931	0.940	4.2	83

^1^ The original YOLOv8n is adopted as the baseline model. ^2^ The original C2f module is replaced by C2f_Star to evaluate the influence of enhanced feature extraction. ^3^ The Spectral-SPPF module is introduced to investigate the contribution of spectral-spatial feature aggregation. ^4^ The complete model integrates C2f_Star, Spectral-SPPF and the optimized detection head.

**Table 2:** Comparison of physical–virtual correspondence performance across 20 scenes.

Scene	Objects	YOLOv8n-seg			RF-DETR + Point Cloud			SF-Lite-YOLOv8n + Point Cloud		
		PE (mm) ^1^	OE (°) ^2^	Latency (ms)	PE (mm) ^1^	OE (°) ^2^	Latency (ms)	PE (mm) ^1^	OE (°) ^2^	Latency (ms)
S1	5	7.2	5.8	81	2.8	2.1	295	2.1	1.5	136
S2	6	7.8	6.2	83	3	2.3	302	2.3	1.7	140
S3	4	6.6	5.4	80	2.6	1.9	289	1.9	1.3	134
S4	8	9.2	7.3	85	3.4	2.6	316	2.6	1.8	145
S5	7	8.5	6.7	84	3.1	2.4	309	2.4	1.7	142
S6	5	8	6.4	82	3	2.2	301	2.2	1.6	138
S7	9	10	7.9	87	3.8	2.9	333	3	2.2	151
S8	6	8.3	6.6	83	3.2	2.5	311	2.5	1.8	143
S9	10	9.8	8.7	89	4.2	3.2	346	3.3	2.4	156
S10	7	8.8	6.5	84	3.3	2.5	319	2.6	1.9	144
S11	8	9.6	7.6	85	3.6	2.8	326	2.8	2	148
S12	5	7.1	5.7	80	2.5	2.1	287	1.8	1.2	133
S13	6	8.4	6.7	82	3.1	2.3	304	2.3	1.6	140
S14	11	11.7	9.2	91	4.5	3.5	356	3.5	2.6	160
S15	9	10.4	8.2	88	3.9	3	339	3.1	2.2	153
S16	7	9.1	7.1	84	3.4	2.6	316	2.5	1.8	144
S17	12	12.5	9.8	94	5	3.9	371	4	2.9	168
S18	8	9.8	7.8	86	3.7	2.8	331	2.9	2.1	149
S19	10	11.2	8.9	90	4.3	3.3	349	3.4	2.5	157
S20	6	7.9	6.3	82	3	2.2	301	2.2	1.6	138
Average		9.1	7.2	85	3.5	2.7	320	2.7	1.9	146

^1^ PE (mm): Position Error, calculated as the Euclidean distance between the reconstructed and reference positions. ^2^ OE (°): Orientation Error, calculated as the equivalent rotation angle between the reconstructed and reference rotation matrices.

## Data Availability

The data supporting the reported results include a publicly available benchmark dataset and self-collected experimental data. The MVTec ITODD dataset analyzed in this study is publicly available from its original source. The self-collected manufacturing scene data used for the physical–virtual mapping experiments are not publicly available due to applicable restrictions but may be obtained from the corresponding author upon reasonable request.

## Abbreviations

The following abbreviations are used in this manuscript:

SF-Lite-YOLOv8n	Structure-aware Feature Lightweight YOLOv8n
YOLO	You Only Look Once
SPPF	Spatial Pyramid Pooling Fast
DCT	Discrete Cosine Transform
C2f	Cross Stage Partial with two feature fusion
Detect_Efficient	Efficient Detection Head with Shared Feature Encoding
mAP	mean Average Precision
GFLOPs	Giga Floating-point Operations
FPS	Frames Per Second
